# On Curating Multimodal Sensory Data for Health and Wellness Platforms

**DOI:** 10.3390/s16070980

**Published:** 2016-06-27

**Authors:** Muhammad Bilal Amin, Oresti Banos, Wajahat Ali Khan, Hafiz Syed Muhammad Bilal, Jinhyuk Gong, Dinh-Mao Bui, Soung Ho Cho, Shujaat Hussain, Taqdir Ali, Usman Akhtar, Tae Choong Chung, Sungyoung Lee

**Affiliations:** 1 Ubiquitous Computing Lab, Department of Computing Engineering, Kyung Hee University, Giheung-gu, Yongin-si, Gyeonggi-do, Seoul 446-701, Korea; mbilalamin@oslab.khu.ac.kr (M.B.A.); wajahat.alikhan@oslab.khu.ac.kr (W.A.K.); bilalrizvi@oslab.khu.ac.kr (H.S.M.B.); kjh@oslab.khu.ac.kr (J.G.); mao@oslab.khu.ac.kr (D.-M.B.); chosoungho@oslab.khu.ac.kr (S.H.C.); shujaat.hussain@oslab.khu.ac.kr (S.H.); taqdir.ali@oslab.khu.ac.kr (T.A.); usman@oslab.khu.ac.kr (U.A.); sylee@oslab.khu.ac.kr (S.Y.L.); 2 Telemedicine Group, Center for Telematics and Information Technology, University of Twente, Enschede 7500AE, The Netherlands; o.banoslegran@utwente.nl; 3 Intelligent System Laboratory, Department of Computer Engineering, Kyung Hee University, Giheung-gu, Yongin-si, Gyeonggi-do, Seoul 446-701, Korea

**Keywords:** data curation, multimodal sensory data, data acquisition, lifelog, healthcare, wellness platform

## Abstract

In recent years, the focus of healthcare and wellness technologies has shown a significant shift towards personal vital signs devices. The technology has evolved from smartphone-based wellness applications to fitness bands and smartwatches. The novelty of these devices is the accumulation of activity data as their users go about their daily life routine. However, these implementations are device specific and lack the ability to incorporate multimodal data sources. Data accumulated in their usage does not offer rich contextual information that is adequate for providing a holistic view of a user’s lifelog. As a result, making decisions and generating recommendations based on this data are single dimensional. In this paper, we present our Data Curation Framework (DCF) which is device independent and accumulates a user’s sensory data from multimodal data sources in real time. DCF curates the context of this accumulated data over the user’s lifelog. DCF provides rule-based anomaly detection over this context-rich lifelog in real time. To provide computation and persistence over the large volume of sensory data, DCF utilizes the distributed and ubiquitous environment of the cloud platform. DCF has been evaluated for its performance, correctness, ability to detect complex anomalies, and management support for a large volume of sensory data.

## 1. Introduction

In recent years, there has been a shift in the way healthcare is handled and its supporting systems. This change has made a drastic impact on the design of conventional healthcare models. Instead of late disease management and cure, these models are focusing on preventative-personalized health. Consequently, an opportunity is provided for healthcare providers to focus on when, where, and how; care and support are delivered to the particular patient and service consumer [1,2]. The reason for this shift is the rising financial stress that healthcare systems have to face to support the growing demand for their services [3]. Therefore, service providers are pushing forward for wellness-based models and conducting research to investigate their effectiveness. The latest studies in biomedical healthcare have shown that the most prevalent diseases are partly caused or aggravated by poor lifestyle choices that people make in their daily routine. Unhealthy and fast-food diets, use of tobacco, and sedentary routines with a lack of exercise are among the potential contributors to developing illnesses and also limit the effectiveness of medical treatments [4,5,6].

With the advent of smart and personal devices, an opportunity has emerged for healthcare providers and biomedical researchers to empower people to take care of their health and wellness by providing them with timely, ubiquitous, and personalized support [7]. Consequently, the influx of fitness wearables with smartphone applications and systems supporting health and wellness has taken the market by storm [8]. For example, commercial systems and platforms such as Withings Activite [9], Garmin Vivofit [10], Fitbit Surge [11], Misfit Shine [12], Apple Watch [13] and HealthKit [14], Samsung Gear [15], LG smartwatches [16], Google Fit [17], and Microsoft Band [18] with Microsoft Health [19], all primarily consist of sensor-based bracelets accompanied by mobile apps, and provide some basic health recommendations based on the measured steps, calories, or hours of sleep. In parallel, research groups are also working on health and wellness systems that can alert of physical conditions [20] or detect chronic illnesses [21]. Despite this enormous effort by the industry and research, most current solutions are single-device focused and have a limited scope [1]. Therefore, they are unable to generate a context-rich user lifelog which can provide a holistic view of user activity and behavior [22]. Such lifelogs are a necessity for the evolutionary wellness systems that support the self-quantification of its users [23]. Moreover, a context-rich lifelog is also a low-cost way to acquire valuable user information on which effective interventions from healthcare professionals can be based [24]. Considering the limitations of existing efforts as an opportunity, we have proposed and implemented a comprehensive cloud-based sensory data acquisition framework called Data Curation Framework or DCF.

**Figure 1:**
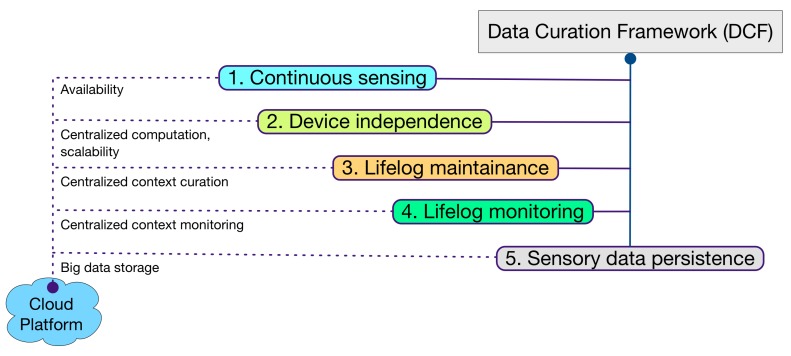
Data curation framework (DCF) philosophy.

The contribution of Data Curation Framework (DCF) is aligned with the definition of lifelog, meaning it is a black box of user life events [22]. Multidimensional insights into user activities and behaviors require a context-rich lifelog that can be developed by the accumulation of data from a larger set of multimodal data sources [22,25]. Keeping this perspective as the primary motivation, DCF implements five core concepts in its design philosophy (illustrated in Figure 1). (i) The ability to continuously sense for raw sensory data from multimodal data sources in real time; (ii) The device-independent acquisition of raw sensory data. This concept contributes to the compatibility of DCF for IoT-based environments [26]. This property increases the ability of DCF to integrate a larger set of sensory devices; (iii) The induction of a larger set of sensory devices results in a richer context. DCF provides comprehensive curation of this context over a user lifelog. This context-rich lifelog can be used in multidimensional ways, e.g., data accumulated from a smartphone, a smartwatch, and a depth camera can accurately identify the context of a user posture in an environment. Therefore, a health and wellness platform using DCF can make recommendations not only pertaining to his activity but also the later on effect of that activity on his muscular health; (iv) DCF is equipped with a lifelog monitoring tool called LLM. In comparison with device-based activity recognition, lifelog monitoring looks for anomalies over richer context occurring over time. For reliability, expert-driven rules [27] provide intelligence to this monitoring (Rule creation is not part of the DCF scope); (v) DCF provides persistence to support the large volume of heterogeneous and multimodal raw sensory data associated with the lifelog. This property enables DCF to support the forthcoming concepts of data-driven knowledge generation [28], descriptive [29] and predictive analytics [30], and visualization [29].

DCF is a cloud-based implementation, as the cloud supports the infrastructure level DCF requirements. For continuous sensing, the ubiquitous nature of the cloud provides DCF with the ability to acquire sensory data in different contexts and environments. Similarly for device independence, the cloud provides a central yet scalable computational resource that can accumulate sensory data from clients without being concerned with their computational abilities. For lifelog maintenance and monitoring, the cloud provides a hub for context curation and monitoring for anomalies detection. Lastly, to support the volume of data accumulated by DCF, the cloud provides a big data platform.

DCF is currently part of the implementation of our ongoing research project called the Mining Minds platform [31]. DCF executes as a separate layer (Data Curation Layer or DCL) at the infrastructure level of the Mining Minds architecture [1]. It accumulates raw sensory data in real time from multimodal data sources and provides curation to the context identified on that data via the Mining Minds Information Curation Layer (ICL). DCL as the implementation of DCF monitors the context for anomalies based on the rules derived by the Mining Minds Knowledge Curation Layer (KCL). Furthermore, it preserves all of the sensory data in big data storage to support analytics and visualization provided by the Mining Minds Supporting Layer (SL). However, in this paper, we only discuss data curation as a framework independent of the Mining Minds platform.

The contents of this paper are as follows. Section 2 describes the related work with respect to DCF. Section 3 provides an overview of the methodology of DCF. Section 4 presents the details of the components participating in the implementation of DCF. Section 5 describes the execution scenarios of DCF in collaboration with a health and wellness platform. Section 6 evaluates various aspects of DCF including its accuracy, performance, scalability, its ability to perform reliable user lifelog monitoring and persistence. Section 7 concludes this paper.

## 2. Related Work

Following the boom of health- and wellness-based systems; lots of systems for sensory data accumulation and analysis have been proposed for the people’s healthcare and wellbeing. For analysis of these systems we have classified them into three different categories: (i) Mobile Health, where implementation is focused on the evolution of smartphones and their embedded sensors; (ii) Wearable Health, where implementation is incorporating wearable bands and smartwatches in combination with smartphone or gateways; and (iii) Data Accumulation and Curation, where the implementation focuses on the accumulated data from variety of sensors for analysis. The categories are discussed in the following subsections.

### 2.1. Mobile Health

In the last decade, the influx of smartphone usage in our daily lives has been enormous. These smartphones are equipment with various multimodal sensors like accelerometer, PPG, and GPS, which in fact used intelligently, can accumulate user activity data in real-time for further utilization. Therefore, the combination of smartphone devices and web services provides more opportunities for healthcare systems to evolve with reduced cost. Researchers have indicated that new requirements and trends are embedded SIM and mobile health platforms (mHealth) [32]. In [33], authors presented the capability of mobile devices (mobility, low-cost, remote, and equipped with sensors) in conjunction with web-service based approach, providing services relating to healthcare. Furthermore, the highlights of the proposed approach are listed and compared with existing remote health monitoring systems. Moreover, in the case of elderly, mobile phones are already participating in providing healthcare services [34]. In [35], the authors presented a mobile phone-based platform to collect the psychological, physiological, and activity information of the user for mental health research. In [36], a mobile version of a data processing toolbox originally devised for computer-based architectures and principally used for human behavior modeling was provided. In [37], a middleware integrating multiple interfaces for multi-parameter monitoring of physiological measurement is proposed. In [38], tools have been suggested for the analysis of the provenance of mobile health data. In [39], authors present a client-server life logging platform. It enables context capturing by the mobile phone and data processing at the server to extract meaningful high-level context.

In [40], the authors present a lightweight lifelogging framework called UbiqLog. This framework provides a configurable interface to enable and disable sensors within the smartphone. Although more sensors can be added due to the compatibility of the framework’s data model; however, the sensors have to be embedded within the form factor of smartphone device. Authors in [40] acknowledge the fact that lifelog data tends to grow rapidly; therefore, an external server like storage is required for permanent persistence. Lifelog generated by UbiqLog is neither shareable or reusable by external systems; furthermore, it does not support any monitoring on the data. Similarly in [41], authors present an open-source toolkit for sensory data collection over smartphones called AWARE. For conducting small-scale studies, AWARE stores all the accumulated data locally on the smartphone; however for larger scale, data is uploaded to the cloud. The toolkit provides a context plugin that can passively perform analysis on the accumulated data. In similarity with DCF, AWARE also implements a publish-subscribe model; however, it is only utilized for context sharing.

In [42], the DigMem system is proposed, which utilizes distributed mobile services, linked data, and machine learning to create rich and interactive HDMs (Human Digital Memory). An HDM, created by the data from the pervasive devices available in user’s environment, produces a more dynamic and data-rich memory. Information such as how the user felt, where the user was, and the context of the environment can be established [42]. In contrast with DCF, the DigMem is a tri-component system with no service-based support for sharing its core asset data, i.e., memory boxes with health and wellness platforms. Although it collects data from multimodal sources, DigMem requires a compatible environment where its smartphone application can broadcast and look for information. Collected data is linked using semantic web technologies and presented as a memory box to its web application for visualization. DigMem’s concept of linked data is conceptually similar to DCF’s curation of user context; however, the focus of DigMem is on the visualization aspect of this data. Whereas in DCF, curated data is monitored for anomalies and its services provide lifelog as a user timeline for visualization. In [42], authors acknowledge the inclusion of big data in lifelogging systems and discusses the challenges involved in its incorporation in [43]. However, the implementation of big data in DigMem is not addressed.

### 2.2. Wearable Health

In the last decade, the usage of wearables has evolved from medical needs to a personalized accessory. Projects like SenseWare(SWA) [44] armband that collects physiological data using a bi-axial accelerometer, galvanic skin resistance (sweat rates), heat flux (heat dissipated from the body), and skin and near body temperature, have been utilized. In [45], the authors used SWA for the estimation of energy expenditure and step count during treadmill walking in cystic fibrosis patients, compared to healthy adults. In [46], the SWA has also been utilized for the monitoring of adherence in women with rheumatoid arthritis.

In the wearable health research Microsoft’s SenseCam [47] is considered a revolutionary pervasive device [48]. It is equipped with a digital camera and multiple sensors, including sensors to detect changes in light levels, an accelerometer, a thermometer for detecting ambient temperature, and a passive infrared sensor for detecting the presence of people. SenseCam has been used in various studies, for example, in [49], authors use the technology as a memory aid for capturing the user’s daily routine. The images recorded are mapped as a lifelog and presented in a timeline format. In [50], the authors use SenseCam for the tracking of sedentary behavior. However, the focus of this utilization is about activity data accumulation and tracking.

In [51], authors present the InSense system. It utilizes acceleration, audio, and visual sensing, to perform real time context recognition. Conceptually InSense and DCF can be aligned and compared as they both work for real time data collection with a larger set of data sources to generate a context-rich lifelog. However in InSense, data accumulation and its understanding are two separate activities. After the data is collected, it is manually annotated and rated by the user to create an interest operator. This process is completely offline and quite cumbersome for the user who has recorded an activity for few hours. Furthermore, the lifelog becomes rich as a consequence to annotation; thus, an effective monitoring in real time cannot be performed for the InSense users.

In more recent years, the utilization of multimodal sensor-based technologies has evolved to become personalized accessories (e.g., wearable bands and smartwatches such as Garmin Vivofit, Fitbit Surge, Misfit Shine, and Apple Watch). These technologies process independently of smartphones. The wearable wrist sensor stores and transfers the sensory data using a dongle device or a smartphone to a health management system. However, these systems are single data source-oriented, i.e., they only consider a wristband sensor; therefore they cannot infer correct context about what a user is presently doing.

Regardless of the considerable research and development of healthcare and wellness systems, only a few implementations exist that execute as independent platforms for tackling complex and realistic scenarios. Highly funded commercial initiatives like Apple’s HealthKit, Google Fit, and Microsoft Health have evolved the approach from an application to an ecosystem; however, these implementations are still single device or data integration-centric.

### 2.3. Data Accumulation and Curation

Considering lifelog data as an asset, some of the latest works have been focused on data accumulation and its extended utilization. In [52], the authors present the challenges of data accumulation in the Internet of Nano-Things [53] (IoNT) perspective. The challenges are discussed at communication protocol level, hardware level and software (middleware) level. Possible solutions and applications have been discussed as a proposed design.

In [54], authors present an energy efficient continuous sensing framework called Insight. The implementation of this framework focuses on small and wearable devices, and has been demonstrated using a smartwatch. The data accumulation process is energy efficient, and this data is further used for prediction. Comparing the implementation of Insight with DCF, it is evident that the volume of data both the frameworks are concentration on is very different. Insight only uses wearable devices with small data footprint; furthermore, it does not use raw sensory data. If required, it leverages secondary application like Google Fit for the user activity data. Opportunistic (Event and interval driven) sensing [55] of Insight with stated sensing policy also contributes to limited size. This data is persisted in a folder like structure on the device’s storage. On the other hand, DCF implements continuous accumulation in a device-independent mode; resulting in a much more scalable framework for a larger set of multimodal data sources. Consequently, the data generated in DCF is significant in volume which requires big data storage and the curated lifelog is context rich. DCF provides services on sharability of this data for knowledge generation, analytics, and visualization. As acknowledged by the authors in [54], computationally complex processes should be handled by devices with advanced computing abilities. Therefore, DCF utilizes the computational ability of the cloud to execute compute-intensive processes on the accumulated raw sensory data.

Some of the systems have a particular perspective on the accumulation and utilization of healthcare data originated at the clinical level. Instead of more real-time accumulation, these implementations are focused on evolutionary repositories with aggregation of historical and more recent clinical data available for utilization. Among these clinical data-oriented systems, NetBio [56] is a prominent implementation. It assembles vast amounts of curated and annotated, clinical and molecular data. This method enables NetBio clients to make unique discoveries that otherwise would be impossible with their own private datasets. NetBio uses big data technology for permanent persistence and core logic layers to make correlations between the billions of data points from the public domain with private genomic and clinical data sets. At a higher-level, NetBio provides a rich set of APIs that enable clients to integrate NetBio within their workflows and scenarios. Their current clients include pharmaceutical R&D and academic medical centers. The initial system was implemented for oncology; nevertheless, it is now expanding into metabolic and autoimmune diseases.

In contrast to all of the implementations discussed above, DCF is a novel attempt to implement a raw sensory data acquisition, curation, and monitoring framework. The sensory data acquisition services of DCF are independent of data sources. It is equipped to handle multimodal data sources directly communicating with the framework or via smartphone-like gateways. The acquired multimodal data is synchronized to represent an event of time for each user. With scalability in mind, numerous multimodal data sources can communicate in parallel with the DCF, making it a more IoT-oriented implementation. Furthermore, instead of being dependent on the computational ability of smartphones, DCF considers all of the communicating devices as a source of raw sensory data; thus, generating a context-rich user lifelog. The computation over the accumulated data and lifelog is performed over a cloud platform, keeping the framework compatible with data source with low computational abilities. From an evolutionary perspective, complex computational algorithms for context identification, data fusion, and mining can be implemented without disturbing client implementations. Moreover, the accumulated data can be used for concepts of data-driven knowledge generation, descriptive and predictive analytics, and visualization. Samsung SAMI [57] is also moving in a similar direction with an independent API and cloud support; however, their implementation is more data exchange-centric, and utilization for healthcare and wellness requires custom implementation and monitoring. Moreover, DCF curates the identified low- and high-level context on user accumulated data as a comprehensive lifelog. The idea of lifelog has already been presented in [22,24,25]; however, DCF implements anomaly detection on lifelog instances based upon expert-driven rules in correlation with user profiles, keeping the monitoring vigilant as well as personalized.

## 3. Proposed Methodology

This section provides an overview of the proposed methodology of DCF. Participating components in the implementation of DCF are introduced in this section; however, technical details of these components are described in the following section.

DCF is intended to be incorporated as a foundation layer for health and wellness platforms where real-time multimodal sensory data acquisition, its curation as a lifelog, and monitoring for anomalies is essential. Furthermore, it provides permanent storage to acquired sensory data for extended usage (e.g., visualization, analytics, data-driven knowledge generation). For the independent execution of DCF, its implementation is encapsulated with interfaces for sensory data acquisition and its persistence. The curation of a lifelog and its monitoring is implemented as an implicit property of DCF with a separate interface to incorporate anomaly detection rules and publish-subscribe based response.

**Figure 2:**
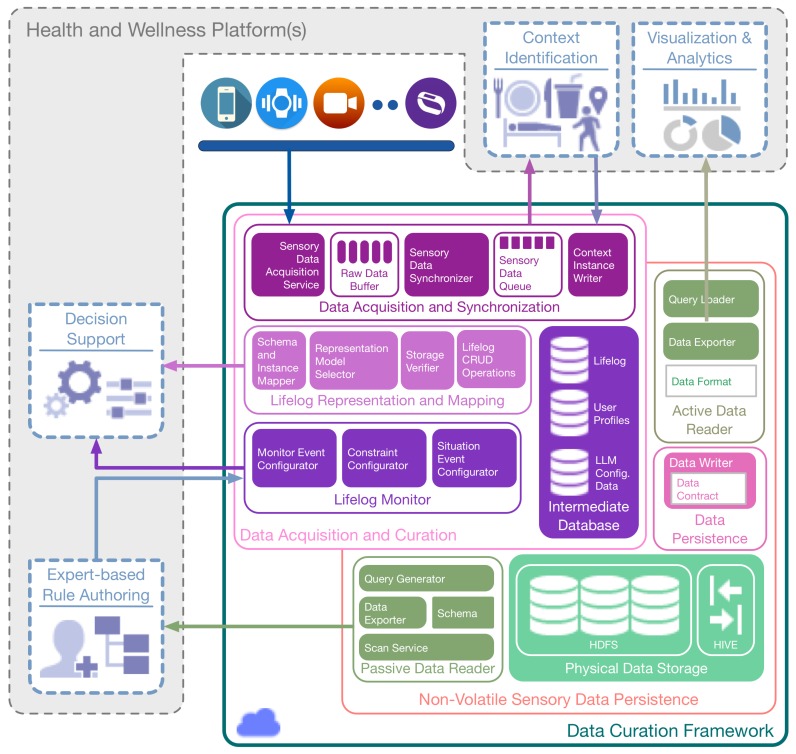
Data curation framework (DCF) architecture.

As illustrated in Figure 2, DCF consists of two primary components, i.e., *Sensory Data Processing and Curation*, and *Non-volatile Sensory Data Persistence*. Within the former, the *Sensory Data Acquisition and Synchronization* subcomponent obtains the raw sensory data from multimodal data sources, both in a real-time (active) and offline (passive) manner. This data is synchronized based upon the user identification and the time stamp of the data generation, and subsequently, it is queued for the context determination. The definition and methodology of the identification of context vary in health and wellness platforms [58]; therefore, an interface to the synchronized sensory data residing in the queue is provided by DCF. This feature facilitates health and wellness platforms to plug-n-play their context identification engine without worrying about the real-time data acquisition from multimodal sources in a distributed environment. In response, these engines can feedback the DCF with identified context. The subcomponent of *Lifelog Mapping and Representation* receives the identified context and curates it by mapping the context instances to a time-based log registering the detected human activities and behaviors. The lifelog persists in the Intermediate Database for reuse. The stream of lifelog instances is analyzed by monitoring the subcomponent known as the *Lifelog Monitor (LLM)*; it is responsible for performing customized unit-based (e.g., time-based, physical activity-based, nutrition-based) monitoring of user context available in the lifelog, cross-linked with the user profiles. *LLM* draws the association between the context available in the lifelog and implements anomaly detection based on expert-driven rules. Anomalies detected by *LLM* represent risky or unhealthy behavior and are described by various constraints (e.g., age, gender, medical conditions) and monitor-able variables (e.g., the intensity of a particular activity and its duration). *LLM* is equipped to provide a notification-based response to its client (a health and wellness platform or a user) with the help of a publish-subscribe mechanism.

The *Non-volatile Sensory Data Persistence* component is responsible for providing permanent and distributed big data persistence to the raw sensory data. It is termed non-volatile, as no update or delete operations are performed on the raw sensory data storage. The subcomponent of *Data Persistence* provides a data contract that is implemented by the clients of *Physical Data Storage* for permanent persistence of the data. *Non-volatile Sensory Data Persistence* also provides mechanisms to access this persisting data as a response to health and wellness platforms. This response can be of an online or offline type. For an online response, the *Active Data Reader* subcomponent is used. It provides a continuous stream of sensory data for extended data operations, including visualization and predictive analytics. For an offline response, the *Passive Data Reader* subcomponent is used. It provides a batch response that can be effectively used for training machine learning-based models and can provide data insights to experts for rule generation. To create periodic backups of lifelog data, the subcomponents of *Data Persistence* are again utilized. This method provides permanent storage of the lifelog data, which can be utilized in the future for user behavior analysis.

## 4. Implementation Details

DCF is a hybrid cloud implementation with components distributed between a private and a public cloud. Therefore, privacy and security of user lifelog data has been given high importance. For its implementation, we have used custom built Health Fog framework [59], which provides Fog computing [60] as an intermediary layer between the client and DCF. Furthermore, it implements Cloud Access Security Broker (CASB) for data access policy. The modular design of Health Fog is capable of engaging data from multimodal data sources together with the adequate level of security and privacy using existing cryptographic primitives.

In the staging and production environments of DCF, the component of Sensory Data Processing and Curation is deployed over a public cloud (Microsoft Azure [61]) with compute-intensive instances to support scalability and high performance. However, the component of Non-volatile Sensory Data Persistence is deployed over a private cloud environment with distributed commodity machines, customized security [62], and large-space hard drives. Implementation details of these components are described in the following subsections.

### 4.1. Raw Sensory Data Acquisition and Synchronization

Implementation of Raw Sensory Data Acquisition and Synchronization (DAS) consists of a REST [63] service that collects raw sensory data from multimodal data sources. The key in this acquisition is the association of accumulated data with their time of origination. All data sources subsist independently along with independent clocks; therefore, a logical clock is required for identifying the data origination at the same time from multiple sources. Consequently, DAS implements the time frame-based synchronization methods called Complete- and Incomplete-sync.

Complete-sync is the sunny day scenario in which all the data pertaining to an instance of time is accumulated within the specified time frame. It waits for the data accumulation from all the data sources at the DCF server within the duration of a three second time frame. As soon as all the data is received, complete-sync encapsulates it as one message. Subsequently, the message is timestamped and enqueued in Sensory Data Queue for context determination by a health and wellness platform.

In contrast to Complete-sync, Incomplete-sync executes when data from any of the sources is not received in the three-second time frame. This execution is further classified in two different modes, i.e., Eager and Rendezvous. In the Eager mode, the message is created with only the available data and buffered for context determination. In the Rendezvous mode, for the missing data, it associates the last sent data from the raw sensory buffer. Subsequently, a message is created and buffered for context determination. Implementation details of Complete-sync and Incomplete-sync with its dual modes are explained in Algorithm 1.

Due to the non-blocking (asynchronous) and scalable nature of data acquisition from multimodal data sources, DAS uses Node.js [64] for server-side implementation. Furthermore, it implements in-memory buffers for temporary storage of data that has arrived. Each buffer represents a particular type of multimodal data source. A buffer synchronizer is executed so that the accumulated data can be synchronized with the data that originated at the same time from all other data sources.

For communication from a multimodal data source to DAS, a JSON [65] object is defined. The contents of this object consist of four fields, i.e., a user id, a data source id, a timestamp of the data origination at a data source, and the payload. Due to service-based implementation, DAS is independent of the number of multimodal data sources.

**Algorithm 1** Time-based synchronization for raw-sensory data acquisition.**Require:**
$buffer_{src}\left[1,...,n\right]$: n is the total number of data sources**Ensure:**
$buffer_{dst}$: queue for time-synchronized data packets  1: **procedure** Sync($buffer_{dst}$)  2:  
msg←create_msg(NULL)  3:  **while**
i≤No_of_datasources
**do**  4:   
$buffer_{src}\left[i\right]\leftarrow Recv\left(data\right)$        ▹ Complete-sync execution  5:   
$msg.add(create\_msg\left(buffer_{src}\left[i\right]\right))$  6:   **if**
$time_{sec}>time\_window$
**then**  7:    **if**
send_only=TRUE
**then**      ▹ Incomplete-sync: Eager execution  8:      
break  9:    **end if** 10:    **while**
j≤No_of_datasources
**do**    ▹ Incomplete-sync: Rendezvous execution 11:      
j←i+1 12:      **if**
$buffer_{src}\left[j\right].has\_contents$
**then** 13:        
$msg.add(create\_msg\left(buffer_{src}\left[j\right]\right)$ 14:      **end if** 15:     **end while** 16:    
break 17:   **end if** 18:  **end while** 19:  
$msg.timestamp\leftarrow buffer_{src}\left[i\right].timestamp$ 20:  
$buffer_{dst}.enqueue\left(msg\right)$ 21: **end procedure**

### 4.2. Data Persistence

The asset of DCF is its persistence of a user’s raw sensory data (from multimodal data sources) with the associated context as a lifelog. DCF defines two levels of abstraction on the data acquired. The first level is a higher-level abstraction referred to as an Intermediate Database hosted within a relational database (RDBMS). This database hosts three types of data, i.e., the user lifelog that represents user context over a period of time, the user profiles, and the knowledge base consisting of rules for anomaly detection required by LLM. The intermediate database is deployed over a public cloud instance.

The second level is a lower-level abstraction referred to as non-volatile distributed storage; it is hosted over Hadoop-based big data storage [66] on a private cloud instance. This storage also hosts three types of data; however, the granularity of data is at a finer scale. For example, this storage provides permanent persistence to all the raw sensory data acquired from the multimodal data sources; it maintains user-invoked backup of large-sized multimedia content, such as video data captured from a 3D camera and periodic backups of user lifelogs with associated user profiles.

The philosophy behind this two-level abstraction architecture of data storage is due to four main factors. (i) Performance, the user lifelog data is frequently accessed in soft-real time. Therefore, due to the inherent speed of the RDBMS, its storage is leveraged for lifelog persistence and access; (ii) Data relationships, the user activities and behaviors are encapsulated as context by a comprehensive object-based relationship. For recommendation generation or high-level context determination by health and wellness platforms, relationships among entities of user profiles and their context are necessary. Thus, a tightly coupled structured data formation is held. Therefore, the schema-based approach of the RDBMS is leveraged for persistence instead of the unstructured data storage of big data; (iii) Data volume, the magnitude of data generated by multimodal data sources, grows exponentially in a short period of time. Therefore, big data storage with its distributed non-volatile persistence is utilized for storing the raw sensory data as well as periodic backups of user profiles and lifelogs; (iv) The frequency of data updates, the user lifelog changes and evolves rapidly due to the continuous change in user context in real time. Due to the higher cost of data updates at the big data level, RDBMS is leveraged.

### 4.3. Lifelog Representation and Mapping

**Figure 3:**
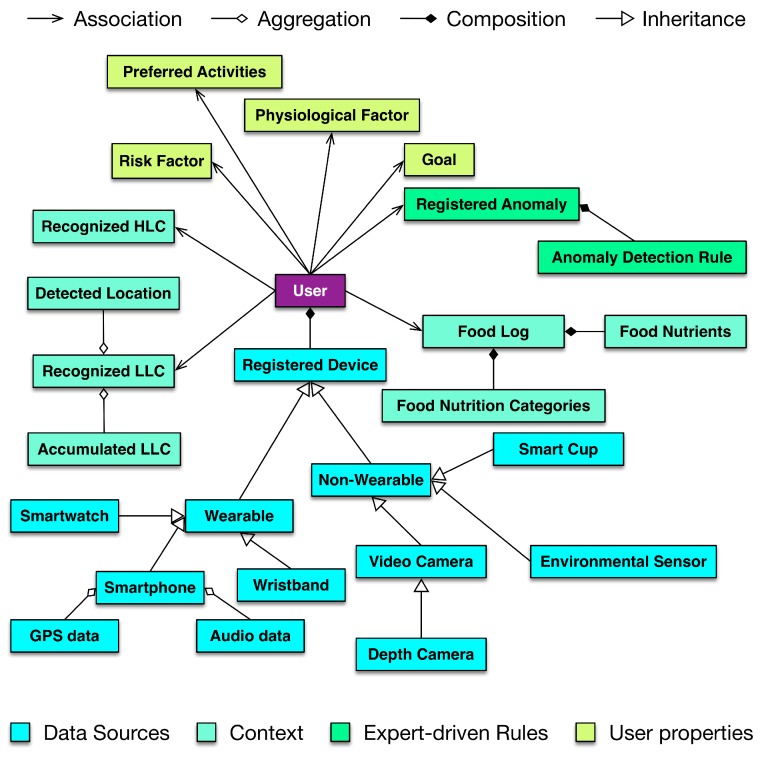
Lifelog model.

In DCF, the identified context is mapped over a timeline as a lifelog, providing a black box to user activities and behaviors over time. The lifelog is maintained in a relational database instance at the intermediate database of DCF. The maintenance of this lifelog including CRUD operations is the responsibility of the subcomponent of Lifelog Representation and Mapping (LLRM). The lifelog is represented as an object-oriented abstraction called a lifelog model in this mapping subcomponent. The lifelog model encapsulates attributes such as performed activities, preferred and visited locations, health and wellness goals to achieve, and recognized emotions of the users. For agility and extensibility, this object model is an implementation of Facade and Adapter design patterns [67]. The conceptual model of LLRM is illustrated in Figure 3.

The implementation of LLRM contains a mapper module that extracts objects, attributes, and relationships from input XML/JSON data as resources. It identifies and maps the classes for the extracted resources and places the instances accordingly using a deep copy method [68].

The Storage Verifier module validates the data according to the schema of the data tables in the intermediate database. Furthermore, it also validates the compatibility of data with the defined constraints (keys, values, primary keys, and foreign keys) on the data tables.

Depending upon the utilization, the lifelog model has higher-level abstractions, called representations, to customize the lifelog data access for health and wellness platforms. The current implementation provides four different representations: (i) The first representation provides an interface to internal DCF components for storing and retrieving lifelog data; (ii) the second representation provides an interface to external clients of DCF for retrieving lifelog data with user profiles; (iii) the third representation provides an interface to external clients of DCF for retrieving lifelog data and its associated raw sensory data from non-volatile storage; (iv) the fourth representation provides an interface to external clients of DCF for incorporating decisions made by health and wellness platforms as part of lifelog data. These representations are compatible with XML and JSON document formats.

### 4.4. Lifelog Monitoring

Lifelog Monitoring (LLM) is designed to perform continuous monitoring of the user context representing his activities and behavior in his lifelog. LLM itself does not recognize activities; however, it leverages the external activity recognition abilities of the health and wellness platforms to provide associated context, and it executes at a higher-level abstraction and draws associations between a user’s low- and high-level context [69], then maps them to the anomaly detection rules built by an expert. As soon as this mapping is established, LLM registers them over the target user’s lifelog and starts the monitoring process.

To generate notification-based responses, LLM implements an observer pattern [67]. This mechanism facilitates LLM to host a subscriber list of clients for a particular user’s lifelog. If an anomaly is detected, all of the subscribed clients are notified. This implementation is helpful for scenarios in which not only the user but also his care provider and a third party wellness platform need to be alerted to a particular anomaly-related event.

LLM is designed to incorporate anomaly detection rules that are generated by the expert dynamically. This nature of the LLM requires a robust model to handle all of the anomalies to be detected in a particular domain. Therefore, LLM divides domains in terms of measuring quantities. The current implementation of DCF supports time as the measuring quantity for physical activities, as well as macronutrients (fat, carbohydrates, and proteins) and calories for food intake.

The overall complexity of the implementation of LLM is divided into three modules, i.e., the monitored event configurator, the constraint configurator, and the anomaly event detector. Configurator modules are built to incorporate the anomaly detection rules. These rules are submitted to LLM using a JSON-based communication contract. This contract is described in the following listing:

{
// Common communication format
 "AnomalyConditions":[
 {
     "ConditionKey" : "Age",
     "ConditionType" : "String",
     "ConditionValue" : "Adult",
     "ConditionValueOperator" : "="
 },
 {
     "ConditionKey" : "Duration",
     "ConditionType" : "String",
     "ConditionValue" : "1h",
     "ConditionValueOperator" : "="
 },
 {
     "ConditionKey" : "CurrentContext",
     "ConditionType" : "String",
     "ConditionValue" : "Sitting",
     "ConditionValueOperator" : "="
 }, "AnomalyID" : "1"
 ]
}

To uniquely identify dynamic anomalies in multi-situation scenarios, they are managed as key-value pairs. The output of the configurator modules is well-defined anomaly detection information, consisting of its monitored event and its target measuring quantity.

**Figure 4:**
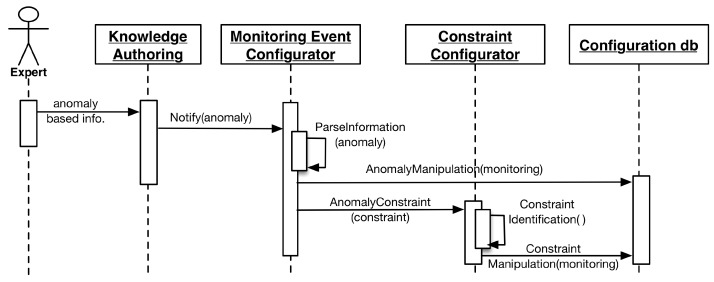
Sequence diagram of anomaly registration by the expert.

**Figure 5:**
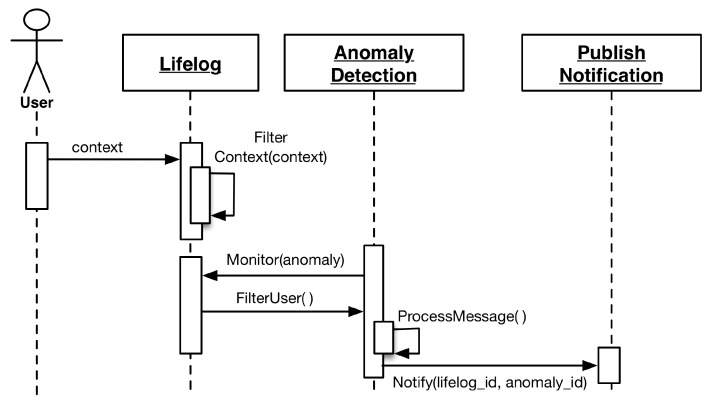
Sequence diagram of message flow in the lifelog monitoring.

The anomaly detector module is responsible for monitoring the user lifelog for monitored events. For monitoring to occur, at least one respective anomaly associated with a particular monitored event must be registered for a user. As soon as the monitored event is detected, the anomaly detector registers the respective well-defined anomaly detection rule for that particular user. Lifelog data pertaining to these monitored events are filtered out from the stream of user context and managed in a monitorable log. This log has information related to the target measuring quantities. Different monitored events have different target quantities; therefore, the monitorable log facilitates the LLM to manage multiple monitored events for independent monitoring by persisting unique targets. The anomaly detector compares the difference of the measured quantities in regular intervals. Whenever the target is achieved, the anomaly detector publishes a notification to all the subscribers of the user’s lifelog. The execution process of LLM is illustrated in the sequence diagrams of Figure 4 and Figure 5.

### 4.5. Non-Volatile Sensory Data Persistence

The component of non-volatile sensory data persistence is responsible for providing permanent and distributed big data storage to raw sensory and lifelog data. Its subcomponents are designed to execute create and read operations as per the needs of the health and wellness platforms. These components primarily use Hive [70] queries over the Hadoop Distributed File System (HDFS).

The subcomponent of Big Data Persistence is responsible for storing the raw sensory data communicated from the data acquisition and synchronization component every three seconds. This subcomponent is also used for user invoked periodic persistence of multimedia contents such as 3D depth videos.

There are two types of data reading subcomponents for access over distributed storage, i.e., Active Data Reader and Passive Data Reader. Active data reader (ADR) is primarily used to provide a continuous response to a real-time request for data visualization and analytics by a health and wellness platform. ADR hosts a repository of pre-defined Hive queries for a data read. It is an evolutionary repository that grows with more usage and the addition of more clients. Upon receiving the request for data, ADR matches the request parameters with the available set of queries. In the case of a parameter match, the selected query is executed with a continuous stream-based response back to the client.

A passive data reader (PDR) is primarily designed from a machine learning perspective, as historical data persistence in the HDFS is useful for the training of models for data-driven knowledge generation. These models can support the expert to generate rules, which can be further utilized by LLM for anomaly detection. Execution of PDR is a two-step process: first, the schema of the data is sent to the client for parameter selection and request generation; second, after the selection is performed and sent to PDR with the request, it generates a query dynamically upon receiving the parameters. This query is executed over HDFS and the response is created and provided in a JSON format. The execution of PDR is an offline process and may take hours to complete.

## 5. Execution Scenarios

**Figure 6:**
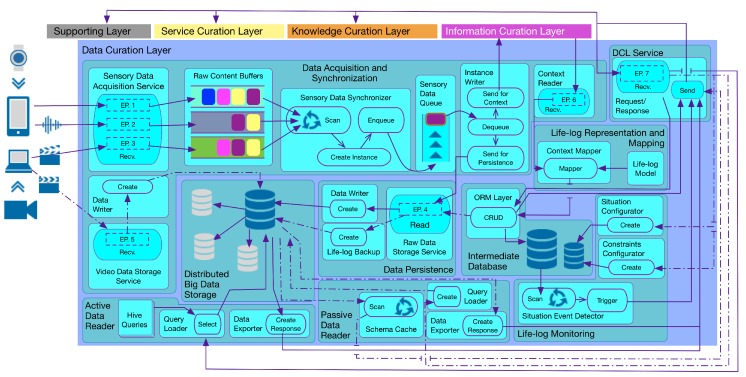
DCF execution flow.

To understand the workings of DCF, we have incorporated its execution in the Mining Minds project as our client health and wellness platform. Depending on the needs of the platform and its participating layers (ICL, KCL, Service Curation Layer or SCL, and SL), DCF concurrently performs the online and offline execution. These executions are termed as active and passive execution flows. These execution flows are illustrated in Figure 6 and described in the following subsections.

### 5.1. Active Execution Scenario

The latest version of the Mining Minds platform supports the recognition of ten activities and three emotions (described in Table 1). They are recognized by ICL and returned as identified high- and low-level context. To facilitate ICL, DCF acquires four different types of raw sensory data (activity, location, audio, and 3D video) per user in an online manner. DCF communicates with three multimodal data sources for raw sensory data acquisition, i.e., a smartwatch, a smartphone, and a depth camera. The smartwatch accumulates accelerometer, gyroscope, magnetometer and PPG data, and synchronizes itself with the Mining Minds smartphone app. The smartphone itself collects accelerometer, gyroscope, magnetometer, GPS, speed, and audio bytes of three seconds for determining user activities, locations and emotions. The Mining Minds smartphone app communicates all this raw sensory data to the DCF. For soft-real-time data acquisition, this non-blocking communication occurs every three seconds. In parallel, a depth camera (Kinect [71]) attached to a PC also communicates with the DCF for sending the semantics of the captured 3D video. These semantics provide activity and behavior-related information of the user to ICL. Inside DCF, the sensory data acquisition service receives this accumulated information asynchronously and buffers the data depending on its data source. These raw data buffers are initialized based on the number of data sources, i.e., each data source has a raw data buffer in the sensory data acquisition and synchronization subcomponent. All the buffer contents are synchronized and enqueued for determination of the associated low- and high-level context. From this point forward, the synchronized data is dequeued and then sent in parallel to ICL and distributed big data storage for context identification and non-volatile persistence respectively.

Upon receiving this synchronized data, ICL identifies the context and communicates it back to DCF. To reduce the ICL to DCF traffic, ICL only communicates if there is a change in context. For example, if a user has been sitting for more than one hour at work, ICL will identify the sitting activity at the office with its low- and high-level context identification. This context will not be communicated to DCF unless the user changes his activity to another. The received context from ICL is converted into a user lifelog instance and mapped to the object-oriented representation of the lifelog by the LLRM subcomponent. Furthermore, this subcomponent is also mandated for executing Create, Read, Update, and Delete (CRUD) operations regularly on lifelog data; therefore, it maintains the mapped data in the intermediate database.

The stream of incoming context is continuously monitored by LLM for anomalies described by well-defined rules. In the case of detection, LLM publishes a notification to SCL of the Mining Minds platform for a possible recommendation generation. Consequently, SCL gathers the required data, including the related context and user profile, and generates and pushes a recommendation to the user’s smartphone. For a user request-based recommendation generation by SCL, DCF operates in a pull-based request response. LLRM receives the query generated by SCL via service. Required data, i.e., user profile and lifelog data, are extracted via a read operation and returned as a response to SCL for recommendation generation.

As mentioned earlier, in parallel to context determination, raw sensory data is also sent to non-volatile storage for persistence. This data is required by SL of the Mining Minds platform for visualization and descriptive analytics. Following a request based on registered parameters, static queries are invoked by the active data reader subcomponent. It generates a stream-based response, as SL requires a continuous stream of data for visualization and analytics.

### 5.2. Passive Execution Scenario

The passive execution flow of DCF pertains to the processes required for evolutionary support of not only DCF but also the overall Mining Minds platform. One of the primary processes that requires the passive execution of DCF is the provisioning of raw sensory data from non-volatile persistence for data-driven knowledge generation by KCL of the Mining Minds platform. This execution is based on a two-step process. First, KCL requests DCF for the schema of data maintained in the distributed big data storage. The passive data reader subcomponent processes this request by replying to KCL with the most recent version of the schema describing the data maintained in big data storage. Consequently, a domain expert at KCL’s authoring tool selects the parameters based on the schema and generates the conditions for which data should be extracted from the big data storage. These parameters with conditions are returned to the passive data reader, which generates a dynamic query based upon the KCL provided parameters and executes it over big data storage. A response to this execution is returned to KCL in a pre-defined service contract. Extracted data from DCF assists KCL in the generation of rules that are used by LLM in the continuous monitoring of lifelog data during the active execution flow.

Another process that invokes the passive execution of DCF is the periodic backup and synchronization of the user’s lifelog data persisted in the intermediate database with the associated raw sensory data maintained in the big data storage. The subcomponent of the lifelog sync performs this operation. SL of the Mining Minds platform uses this historic data for descriptive analytics.

Inside DCF, an instance of the data acquisition service also executes passively to copy the actual video contents from the user’s PC to big data storage. This is also a periodic execution and exclusively authorized by the Mining Minds user.

## 6. Evaluation and Discussion

DCF has been evaluated from various aspects, e.g., from raw sensory data acquisition perspective, its accuracy of synchronization for sensory buffers, performance, and scalability has been evaluated. From context monitoring perspective, the ability of LLM to monitor upon user lifelog for existing anomalies, for dynamically added anomaly detection rules at runtime, and monitoring multiple users with various activities at runtime. From raw sensory data persistence perspective, its ability to read and write sensory data, and execution time of different queries with varying complexities. The results and discussion of these evaluations have been discussed in following subsections.

### 6.1. Accuracy of the Synchronization Process during Raw-Sensory Data Acquisition

**Figure 7:**
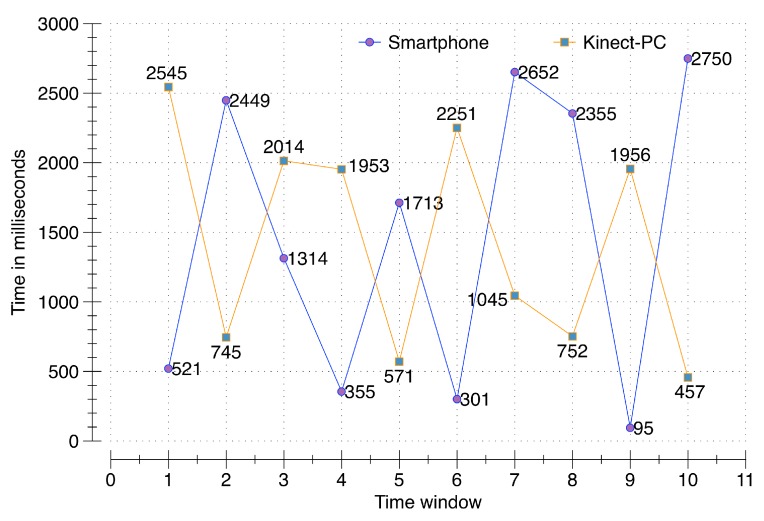
Data synchronization testing per time-window.

To evaluate the accuracy of the synchronization process, a smartphone (Samsung Galaxy S5 running Android 5.0 Lollipop, Samsung, Seoul, South Korea) and second-generation Kinect (rel. 2014) connected to a PC communicate with the DCF server running on a cloud instance with the 64-bit Windows 8.1 operating system, 16 GB of RAM, and a 3.10 GHz AMD A8-7600 Radeon R7, AMD, Sunnyvale, CA USA with 10 computing cores of 4C + 6G. Multiple requests from a single user from both clients (smartphone and PC), generated and synchronized at the server, are validated for accuracy. A time window of three seconds is allocated to temporarily hold the incoming data in memory buffers. For a conclusive evaluation, 100 data packets containing activity, location, voice, and 100 data packets containing video data are sent from the smartphone and the PC respectively. These communications occur randomly at different points in time during the defined three second window. The results from this evaluation reflect that the raw sensory data from both clients has been successfully synchronized at the server. The evaluation in Figure 7 illustrates the exact time in milliseconds of raw sensory data packets sent from both the smartphone and PC during ten different time windows. For example, for the first time window, the smartphone communicated at 521 ms; however, the PC communicated at 2545 ms. The response synchronizer at DCF was able to identify both of these data packets as part of a single window of communication. As illustrated, all of the data packets have been successfully synchronized to the server, proving the correctness of our synchronization process over data acquisition of raw sensory data.

### 6.2. Performance Testing during Raw-Sensory Data Acquisition and Synchronization

**Figure 8:**
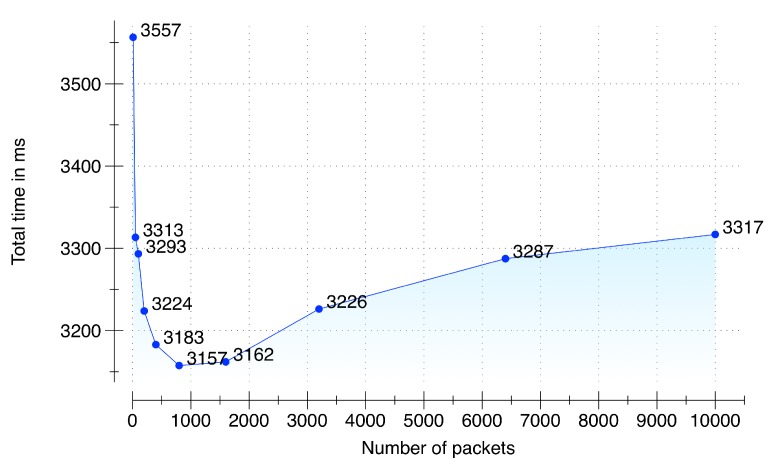
Performance testing.

To evaluate the performance during raw sensory data acquisition and its synchronization we setup an environment of five multimodal data sources: (i) LG Watch R(TM), smartwatch connected with a Samsung Galaxy S5 smartphone running Android 5.0; (ii) Shimmer3 sensor connected with a Samsung Galaxy S5 smartphone running Android 5.0; (iii) Samsung Galaxy S6 Edge running Android 5.0 for audio data; (iv) Second-generation Kinect (rel. 2014) connected with a Samsung Notebook 9 running MS Windows 8.1; and (v) a PC emulating an environmental sensor. These sensors communicate with a DCF server (64-bit Windows 8.1 operating system, 16 GB of RAM, and a 3.10 GHz AMD A8-7600 Radeon R7 with 10 computing cores of 4C + 6G) in an asynchronous manner by sending sensory data packets with an increment from 10 to 10,000. The target timeout deadline given to the data acquisition and synchronization subcomponent is 5 seconds to accumulate, synchronize and queue the synchronized data in a memory buffer. The results of the performance testing are presented in Figure 8. From the results, it is quite evident that our implementation of the data acquisition and synchronization subcomponent is able to accumulate and synchronize 10,000 packets from the five sensors within the provided timeout deadline. Due to server warmup, our implementation initially took 3.5 s for 10 packets; however, the performance improved with subsequent executions. The fastest time was recorded for 800 packets with a total time of 3.15 s. The time to accumulate, synchronize and enqueue increased after 1800 packets and reached at 10,000 packets with a total time of 3.31 s, which is still 51% faster than the provided deadline of 5 s.

### 6.3. Scalability Testing during Raw Sensory Data Acquisition and Synchornization

**Figure 9:**
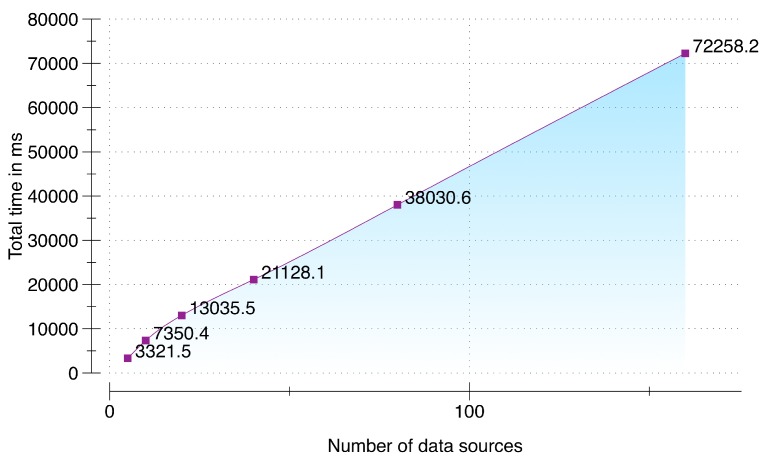
Scalability testing.

**Figure 10:**
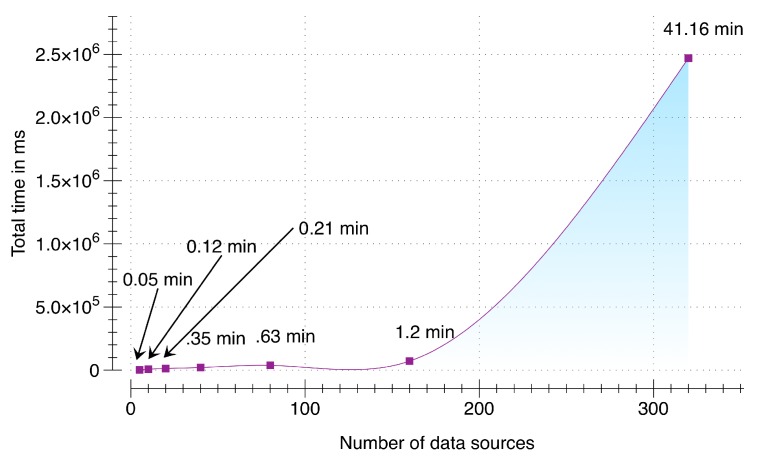
Stress testing of scalability.

To evaluate the ability of DCF to scale appropriately with the increase in number of multimodal data sources we setup an environment that simulates an incremental number of data sources (from 5 to 320). This test case stresses the data acquisition and synchronization subcomponent with a fixed packet size of 10,000 packets per data source with each packet containing a pre-accumulated sensory data of user activity for three seconds (30 kb) by using Samsung Galaxy S5 smartphone running Android 5.0. DCF server is running on a cloud instance with NodeJS v5.8, 64-bit Windows 7 Operating System, 4 GB of RAM, and 3.8 GHz AMD A10-5800K APU. Results of scalability testing are presented in Figure 9. From the results, it can be seen that our implementation was able to scale successfully from 5 multimodal data sources to 160. For 160 data sources with the maximum of 10,000 packets per data source, DCF was able to accumulate, synchronize and enqueue within 1.2 min (=72,258.2 ms). Therefore, our presented implementation can contribute significantly in environments where larger set of data sources will be added over time. As a response, DCF can accumulate, synchronize, and buffer the raw sensory data for these devices without any distinctive time delay. Although this evaluation depends on the size of the data packets; however, from our calculation user activity data accumulated over a duration of three seconds using a smartwatch and a smartphone is 24 kb, which is smaller than the size of the packet used in this evaluation. To check the threshold of our implementation, we stress tested this evaluation with 320 data sources (illustrated in Figure 10). Consequently, the total time increased exponentially to 41 min (=2,470,064 ms).

### 6.4. User Lifelog Monitoring

The presence of an anomaly is a key-driving concept of lifelog monitoring. In the presence of registered anomaly detection rules, LLM provides monitoring for single or multiple users over their lifelog.

**Figure 11:**
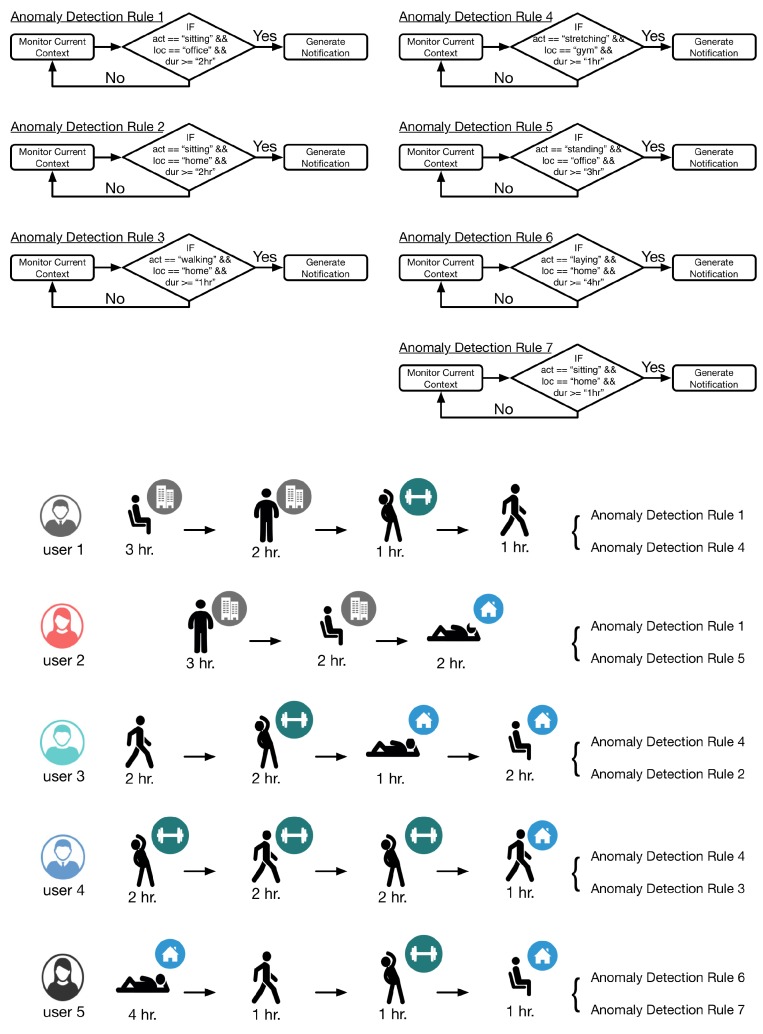
Anomaly detection rules and scenarios.

For the evaluation of LLM, five users were selected to provide context information by performing various activities at user specified locations. Each user provided context using an LG Watch R(TM) for activity data (accelerometer, gyroscope, and PPG sensors were used), smartwatch connected with a Samsung Galaxy S5 smartphone running Android 5.0 for location data (GPS sensor was used) and a Second-generation Kinect (rel. 2014) connected with a Samsung Notebook 9 running MS Windows 8.1 for indoor activity data using depth camera. Using the LLM interface, seven rules were dynamically incorporated in the execution of LLM. Definition of these rules and their correspondence with the users is illustrated in Figure 11. The monitoring was performed over a seven hour snapshot of user lifelog; however, to save the time of the participants, the hour was scaled down to minutes. DCF accumulated 840 records of entries per user (= 4200 records in total); however, as lifelog only records when the context change and marks the enteries with starting and ending time, the number of records vary from user to user.

**Figure 12:**
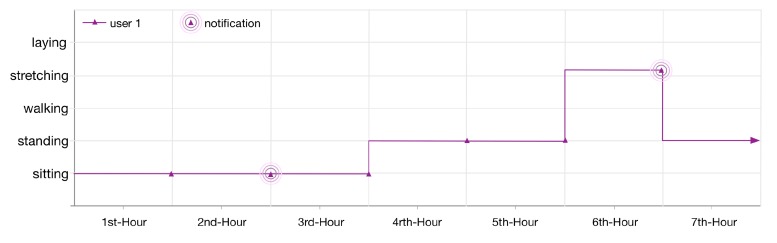
User 1, 7 h lifelog.

The monitored lifelog of the user 1 is shown in Figure 12. The context of the user is sitting at his workplace. After continuously monitoring for 2 h, LLM publishes a notification as he has been in a sedentary state according to the applied anomaly detection rule 1. The user continues his context for another hour then changes it to standing for 2 h and later on doing some exercise at the gym. According to another registered rule for his lifelog, as the user continues the exercise for 1 h, LLM publishes another notification.

**Figure 13:**
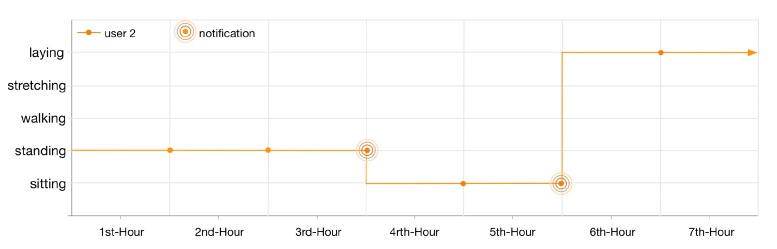
User 2, 7 h lifelog.

Similar to the first user, the lifelog of user 2 is illustrated in Figure 13. As the lifelog of this user is registered with anomaly detection rules 1 and 5; therefore, the user has been notified twice in 7 h. Firstly, for standing at her workplace for 3 h; nevertheless, she continues her context for another hour. Secondly, for being sedentary and sitting at her workplace for continuous 2 h.

**Figure 14:**
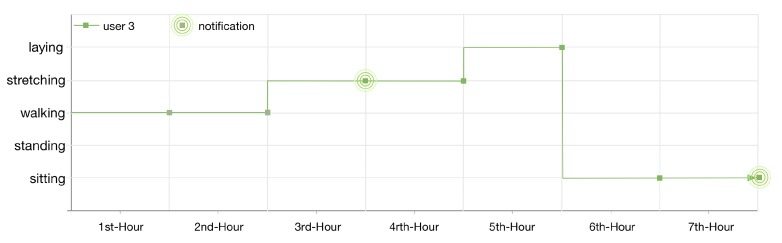
User 3, 7 h lifelog.

The lifelog of user 3 is illustrated in Figure 14. As the lifelog of this user is registered with anomaly detection rules 4 and 2; therefore, the user has been notified twice in 7 h. Firstly, for doing exercise at the gym for continuous 1 h; nevertheless, the user continues his context for another hour. Secondly, for being sedentary and sitting at his home for continuous 2 h.

**Figure 15:**
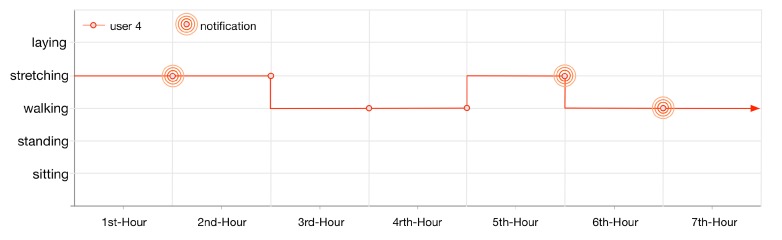
User 4, 7 h lifelog.

User 4 gets notified thrice (illustrated in Figure 15) as his lifelog is registered for anomaly detection rules 3 and 4. These rules are invoked by LLM when the user performs exercise at the gym for continuously 1 h twice and walking at home for 1 h.

**Figure 16:**
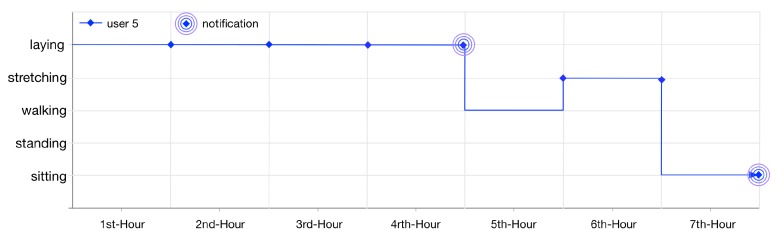
User 5, 7 h lifelog.

User 5 gets notified twice (illustrated in Figure 16) as her lifelog is registered for anomaly detection rules 6 and 7. These rules are invoked by LLM when the user is laying at home for continuously 4 h and sitting at home for 1 h.

The performance of the LLM is based on its timely generation of notifications for the health and wellness services of client frameworks. For its evaluation, the delay between the occurrence of anomaly and publication of notification is evaluated. The results are crosschecked with the help of anomaly log. LLM monitors the lifelog based on a cyclic monitoring interval defined in seconds. It publishes the subscribers with the maximum delay less than the interval of monitoring cycle.

**Figure 17:**
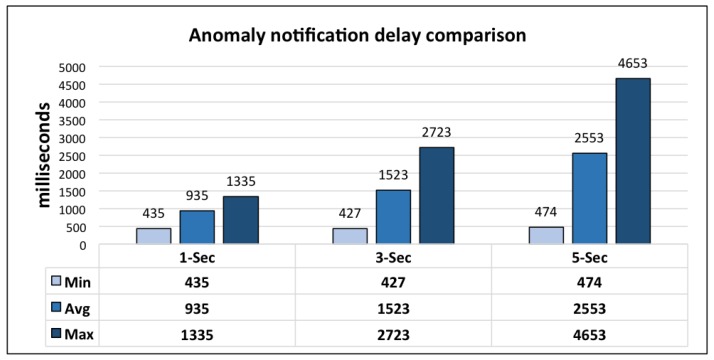
LLM performance evaluation.

For this evaluation 1000 requests were generated for each monitoring interval and DCF server hosting LLM is running on a cloud instance with 64-bit Windows 8.1 Operating System, 16GB of RAM, and 3.10 GHz AMD A8-7600 Radeon R7 with 10 computing cores of 4C + 6G. Results from this evaluation are shown in Figure 17. From these results it can be observed that the interval duration greater than 1 s is appropriate for the evaluation environment, i.e., integrated execution with mining minds platform. Due to the distributed nature of mining minds platform, client communication overhead is introduced; therefore, leading a delay of little over the monitoring cycle interval in some cases. However, keeping the monitoring cycle interval at 3 s, LLM publishes notifications with 96.97% efficiency. In the case of 5 s, notification publication efficiency is 95.84%.

### 6.5. Non-Volatile Data Persistence

The big data storage is deployed over a private cloud instance at the Ubiquitous Computing Laboratory of Kyung Hee University, South Korea [72].

To evaluate the non-volatile data persistence components over HDFS, we have performed experimentation on read and write operations. These operations have been executed for three different raw sensory data sizes, i.e., 1 Gb, 5 Gb, and 9 Gb. This data is distributed over a private cloud infrastructure having four nodes with the following configurations: (i) Name-node, equipped with Intel Core i-5 3.3 GHz, 4 Gb of RAM; (ii) First data node, equipped with an AMD 2.7 GHz, 2 Gb of RAM; and (iii) 3rd and 4th data nodes are equipped with Intel Core i-5 3.3 GHz, 2 Gb of RAM.

**Figure 18:**
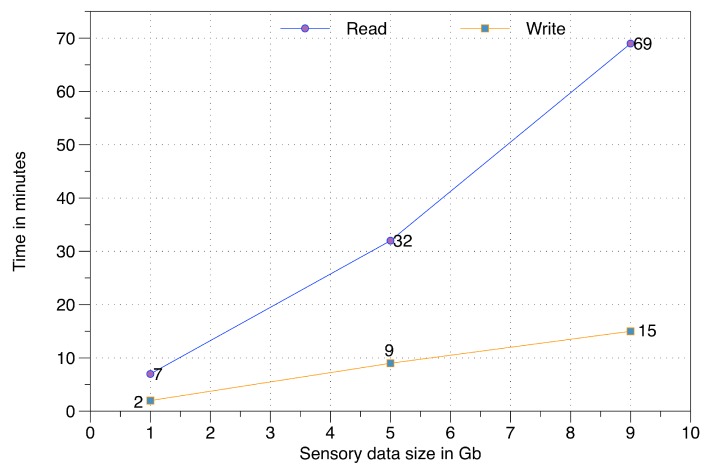
Read and write time over big data storage.

In this evaluation (illustrated in Figure 18), the subcomponents of data persistence and the passive data reader are evaluated. As expected, the write operation is substantially faster than the read operation. The time difference for both read and write is proportional to the volume of raw sensory data in big data storage.

**Figure 19:**
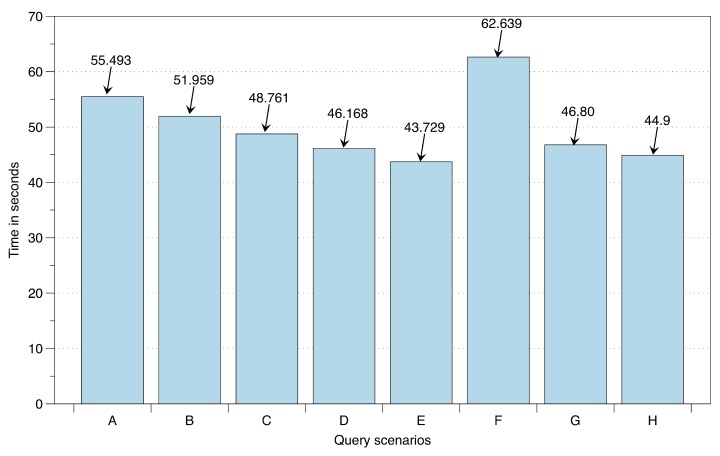
Query execution and response time over big data storage.

To evaluate the execution and response time of the active data reader over HDFS, eight different SL-based queries are executed. These queries (described in Table 2) with varying complexities are performed over 1.7 Gb of lifelog data maintained over big data storage. The structure of this data consists of user detected locations, user recognized low- and high-level context, and record of published recommendations as a response to the anomaly detection. For the user recognized low-level context the data structure consists upon the duration (start and end time) associated with recognized high-level context from identified locations, i.e., Home, Office, Yard, Mall, Restaurant, Outdoors, and Transportation. To measure the response and execution time accurately, each query has been executed 50 times. The evaluation is illustrated in Figure 19. This execution is performed with a single mapper and its associated reducer; however, with an increase in mappers the execution time improves.

From this thorough evaluation, it is evident that DCF can accurately perform synchronization of raw sensory data from multimodal data sources. Its performance-oriented implementation has been validated by performance and load-testing. Its ability to monitor a lifelog has been tested and validated with simple to complex situations for multiple users. Furthermore, the execution-time over data maintained in big data storage has also been evaluated over real raw sensory data sets of large volumes. These evaluations are designed keeping the requirements and contributions of DCF in mind. Furthermore, the results reflect positively on the claimed novelty stated in earlier sections of this paper.

## 7. Conclusions

In this paper, we presented the Data Curation Framework (DCF). This framework focuses on curation of accumulated data from multimodal data sources in real time such that a context-rich user lifelog can be generated. This lifelog offers a holistic view on user activity and behavior which can further be utilized in multidimensional ways including effective interventions from healthcare professionals. The data source-independent implementation of DCF makes it more scalable and IoT compatible. Furthermore, it monitors this lifelog of registered users for the detection of anomalies. This monitoring is able to integrate static, dynamic, and complex created by the expert. DCF incorporates multi-level abstraction on the data, depending upon its usage and persistence. Frequently required user lifelog and profile data is maintained in an intermediate database; whereas, the historic and raw sensory data is maintained in non-volatile storage provided by big data technologies. This property enables DCF to support the forthcoming concepts of data-driven knowledge generation, descriptive and predictive analytics, and visualization.

Keeping the requirements of a data accumulation framework for health and wellness platforms, we have evaluated DCF for its performance, scalability, accuracy of the synchronization process of raw sensory data from multimodal data sources, monitoring of user life log, and data persistence. From the results, it is evident that DCF’s implementation performs efficiently and effectively in realistic situations and scenarios while integrating with a health and wellness platform as the client.

## Figures and Tables

**Table 1:** Multimodal raw sensory data sources.

Data Source	Sensor(s)	Context Type	Context Description
Smartwatch	accelerometer, gyroscope, PPG.	activity	climbing stairs, running, walking, cycling.
Smartphone	accelerometer, gyroscope, longitude, latitude.	location	home, office, gym, school, restaurant, cafe.
Smartphone	audio	emotion	anger, sadness, happiness.
Depth camera	video	activity	eating, sitting, standing, stretching, sweeping, lying down.

**Table 2:** Query execution scenarios.

Scenario	Query
A	SELECT count(userid) FROM userrecognizedactivity WHERE userid = 39;
B	SELECT count(userid) FROM userrecognizedactivity;
C	SELECT count(userid) FROM detected location WHERE StartTime BETWEEN ’1/29/2014’ AND ’12/10/2014’;
D	SELECT count(userid) FROM detected location WHERE LocationLabel = ’Home’;
E	SELECT count(userid) FROM tblUserRecognizedHLC WHERE StartTime BETWEEN ’5/10/2015,2:45:01 AM’ AND ’12/9/2015,11:58:31 PM’;
F	SELECT count(RecommendationID) FROM tfblrecommendation WHERE RecommendationDate BETWEEN ’5/18/2015,10:01:59 AM’ AND ’12/15/2015,2:32:09 PM’;
G	SELECT count(RecommendationFeedbackID) FROM ShetblRecommendationFeedbacket3 WHERE FeedbackDate BETWEEN ’5/18/2015,10:01:59 AM’ AND ’7/29/2015,3:20:28 PM’;
H	SELECT count(UserRecognizedEmotionID) FROM tblUserRecognizedEmotion WHERE StartTime BETWEEN ’5/10/2015,2:30:01 AM’ AND ’12/14/2015,2:00:00 PM’;
